# Porous Gelatin Membranes Obtained from Pickering Emulsions Stabilized with h-BNNS: Application for Polyelectrolyte-Enhanced Ultrafiltration

**DOI:** 10.3390/membranes10070144

**Published:** 2020-07-07

**Authors:** Molka Nafti Mateur, Danae Gonzalez Ortiz, Dorra Jellouli Ennigrou, Karima Horchani-Naifer, Mikhael Bechelany, Philippe Miele, Céline Pochat-Bohatier

**Affiliations:** 1 Institut Européen des Membranes, IEM UMR 5635, Université de Montpellier, CNRS, ENSCM, Place Eugene Bataillon, 34095 Montpellier, France; molkanafti@gmail.com (M.N.M.); danae.gonzales-ortiz@umontpellier.fr (D.G.O.); mikhael.bechelany@umontpellier.fr (M.B.); philippe.miele@umontpellier.fr (P.M.); 2 Physico-Chemical Laboratory of Mineral Materials and their Applications, National Center for Research in Materials Sciences, BP 73, 8027 Soliman, Tunisia; ennigrou2@gmail.com (D.J.E.); karima.horchani@inrst.rnrt.tn (K.H.-N.); 3 Institut Universitaire de France, IUF, 1 Rue Descartes, CEDEX 5, 75231 Paris, France

**Keywords:** hexagonal boron nitride nanosheets, biopolymers, Pickering emulsion, porous membranes, polyelectrolyte ultrafiltration

## Abstract

In recent years, numerous studies have been conducted to develop biopolymer-based membranes, highlighting the challenges to prepare porous structures with control porosity. In this paper an innovative method that relies on the generation of Pickering emulsions was developed to prepare porous membranes from gelatin for filtration purpose. Hexagonal boron nitride nanosheets (h-BNNS) were used to stabilize micro-droplets of castor oil in a continuous homogeneous gelatin solution. Two steps in the membrane preparation process strongly influenced the porous structure. Specifically, the duration of the drying time after emulsion casting and the duration of the cross-linking step affected membrane pore size, hydrophobicity, water swelling, and water permeability. By controlling these two steps, membranes could be designed with pore size between 0.39 and 1.60 μm and display pure water permeability between 150 and 506 L h^−1^ m^−2^ bar^−1^. These membranes have been tested for complexation–ultrafiltration experiments in which iron ions were removed from aqueous solutions with/without poly (acrylic acid) (PAA). Without PAA, the removal of free iron (II) ions was low (not more than 14%). The addition of PAA (200 ppm) allowed obtaining high removal rates (97%) at pH ≥ 5 with 3 bars of transmembrane pressure.

## 1. Introduction

Much research is currently focused on improving wastewater treatment methods to increase the removal of hazardous material, such as heavy metal ions. Membrane technologies are among the most interesting separation techniques for wastewater treatment. For instance, porous polymeric membranes can be used for water filtration [1], pharmaceutical drug purification [2], battery separators [3], and scaffolds for tissue engineering [4].

In the last decades, polymer membranes have attracted major interest for water treatment due to their high energy-saving and pollution control performances [5,6]. However, membrane manufacturing processes have some drawbacks, particularly the use (i) of large quantities of organic solvents that are toxic and the disposal of which is expensive, and (ii) of petrochemical polymers. The growing environmental pollution has been the starting point for researching potential natural polymers able to substitute the conventional ones for membrane preparation. Indeed, porous membranes produced with bio-sourced polymers represent an interesting option due to the use of renewable raw materials. Compared with synthetic polymers, bio-sourced polymers are biocompatible, biodegradable and sustainable. Poly (l-lactic acid) (PLA) is one of most known bio-sourced polymers widely used in health and medical science but recently it has been considered as materials for membranes in connection with water treatment applications. Tanaka et al. produced microfiltration PLA-based membranes using polyethylene glycol (PEG) as pore former via thermally induced phase separation (TIPS) method [7]. These membranes were capable of retaining bacteria cells in a manner of screen filters. In other work, PLA–PEG–PLA triblock copolymers with different PLA segment lengths were used as additive in the preparation of PLA hollow fiber membranes via non-solvent induced phase separation (NIPS). These membranes possess anti-fouling properties towards bovine serum albumin (BSA) about 100% [8]. Wu and Yuan prepared hydrophilic cellulose membranes for gas separation purposes. They measured the permeability of different range of gases (CO_2_, Ar, H_2_, N_2_, CH_4_, O_2_, and He). The membranes were tested both in the dry and water swollen state. When membranes were in swollen state with water, they presented high permeability particularly for CO_2_ [9]. However, many bio-sourced polymers are water soluble [10,11,12] which forces researchers to change paradigm for porous polymeric membrane preparation and requires innovative solutions.

Gelatin is a natural polymer that shows physical-chemical features that might be suitable for the production of dense membranes for gas separation [13,14,15] and of porous scaffolds for tissue engineering [16]. Gelatin presents a complex structure and is produced by controlled hydrolysis of collagen, a fibrous insoluble protein that is the major component of skin, bones, and connective tissue. Due to its many side chains and chemical properties, gelatin is soluble in water. This biopolymer is much used by food and pharmaceutical industries because of its gelling and texturing properties.

New approaches to produce porous membranes with high permeability, good rejection rates and antifouling properties are needed for water purification. The conventional strategies used by membrane manufacturers are based on the so-called “phase separation” technique in which a polymer concentrated solution in a solvent is immersed in a liquid bath (often water) where a polymer-rich and a polymer-poor phase are formed due to the solvent mixing with the liquid [17]. Among the different recent innovative methods for the production of porous polymer materials such as casting technologies, interfacial polymerization, phase inversion, and electrospinning [18,19]. For example, polyacrylonitrile (PAN) porous membranes coated with silver nanoparticles (AgNP) were prepared by electrospinning a PAN solution and functionalized with Ag^+^ by treating them in hydroxylamine (NH_2_OH) aqueous solution. The membranes were tested in terms of water permeability, antimicrobial effect and particulate filtration capability [20]. Novel polysulphone (PS) porous membranes for water desalination, incorporated with carbon nanotubes (CNT), were fabricated by phase inversion method. The membranes present good mechanical properties and high water permeability values about (500 L/h^−1^ m^−2^) when CNT concentrations were 0.5 wt% [21]. Emulsion templates are interesting for the fabrication of water-soluble polymer because they easily allow tailoring the polymer pore size and size distribution. Here, we describe the production of porous gelatin membranes using Pickering emulsion templating to obtain a porous structure. The continuous phase gives the membrane matrix and the disperse phase forms the pores. Pickering emulsions are colloidal emulsions stabilized by solid nanoparticles adsorbed to an oil/water interface instead of organic surfactants. Nanoparticle-stabilized Pickering emulsions are less harmful to the environment compared with surfactants [22] and are more stable. This higher stability is explained by the nanoparticle irreversible adsorption at the liquid–liquid interface that decreases the system energy. Such particles form a rigid shell around the droplets and prevent the emulsion break-up. Once the droplet phase is removed, a spherical void is left in the matrix. Many different materials (e.g., silica [23,24], titania [25,26], alumina [27], iron oxide nanoparticles [28], and two-dimensional materials, such as graphene oxide [29] or hexagonal boron nitride [30,31]) have been employed to produce materials from Pickering emulsions. h-BNNS are particularly interesting because of their high surface area and exceptional features (e.g., stability at high temperatures, increased oxidation resistance, hardness, strong resistance to corrosion, and high thermal conductivity [32]). Moreover, the incorporation of inorganic nanoparticles in the polymeric matrix increases their hydrophilicity and water permeability, and avoids fouling [33].

Here, we describe a new method for preparing h-BNNS/gelatin porous membranes using Pickering emulsions followed by cross-linking with glutaraldehyde (GTA) solution. Changing the curing and cross-linking times allowed producing membranes with various pore size distributions. Then, the cross-linked membranes were tested to assess their stability in water. Pure water permeability measurements confirmed their open porosity.

Conventionally, heavy metal ions are removed and rejected from aqueous solutions by using different methods, such as chemical precipitation, ion exchange, or adsorption. Currently, ultrafiltration, in which a polyelectrolyte is added in the aqueous solutions, is much studied. For instance, in polyelectrolyte-enhanced ultrafiltration (PEUF), large molecular weight polymers are added to the solution where they bind to heavy metal ions that display the opposite charge to form macromolecular complexes. Then, these complexes are trapped and concentrated by ultrafiltration membranes, whereas unbound metal ions are allowed to pass through. Different polymers, such as poly(vinyl alcohol) [34], sulfonated poly(vinyl alcohol) [35,36], poly(ammonium acrylate) [37], poly(acrylic acid) [38,39,40], polyethylenimine [41,42,43,44], and polystyrene sulfonate [45] have been used for metal removal from wastewater. We targeted this application for the as-prepared gelatin membranes to study if they could withstand different conditions of use such as changes in pressure and in pH. This article is divided in two parts. The first part describes the production of porous gelatin membranes using h-BNNS-stabilized Pickering emulsions. In the second part, the iron ion removal efficiency of such totally bio-based membranes was tested as potential application, using poly (acrylic acid) (PAA) as chelating agent. The influence of transmembrane pressure, polyelectrolyte (i.e., PAA) concentration, and pH on Fe (II) ion retention was analyzed.

## 2. Materials and Methods

### 2.1. Materials

Gelatin membranes were prepared using gelatin from porcine skin (CAS No. 9000-70-8, 180 g bloom), castor oil (density = 0.961 g mL^−1^ at 25 °C), GTA (CAS No. 111-30-8, Grade I, 25% in H_2_O), potassium chloride (KCl, CAS No. 7447-40-7, ACS reagent, 99.0%) and ethanol (CAS: 64-17-5, 96%) purchased from Sigma Aldrich (Saint Quentin Fallavier, France). Hexagonal boron nitride (h-BN) (95% purity, 325 mesh, 3 μm particle size) was from Saint Gobain (Courbevoie, France). Two different sizes (0.3 and 1 μm) of polystyrene latex nanoparticles (latex beads) were bought from Thermo Fisher Scientific ( Illkirch, France). Only analytical grade chemicals (without any additional purification) and deionized pure water (18 MΩ) were used for these experiments.

To test iron removal from aqueous solutions by PEUF, PAA with a molecular weight (MW) of 450,000 Da was used. This MW was chosen to make sure that polymers could not pass through the membrane during the filtration process. The iron ion aqueous solutions were prepared with iron chloride (FeCl_2_·4H_2_O) (CAS No. 13478-10-9, 99%) from Sigma Aldrich and ultrapure water produced with a Milli-Q gradient unit (Merck Millipore, Molsheim, France). Hydrochloric acid (CAS No. 7647-01-0, ACS reagent, 37%) and sodium hydroxide (CAS No. 1310-73-2, pellets, >98%) solutions were used to adjust the pH of feed solutions. All chemicals were from Sigma-Aldrich.

### 2.2. Fabrication of Exfoliated H-BNNS

The h-BNNS nanosheets were produced by liquid-phase exfoliation in our laboratory using an ultrasound device (model SONOPLUS HD 3100, 100 W, 20 kHz) and a micro-tip with a 3 mm diameter (MS73). The protocol was described elsewhere [46]. Briefly, after heating the h-BN solution (1 g of pristine h-BN in 100 mL of water) to 80 °C, 20 g of gelatin was added and heated until complete dissolution, and then KCl was added. These ionic species should interpose between the layered h-BN structures to increase the interlayer spacing. The solution was dispersed at 50 °C in a bath to avoid gelatin solidification and was sonicated for 3 h (65% amplitude with pulse off/on 0.5–1 s). Afterwards, the solution was centrifuged twice, first at 3000 rpm for 30 min and then at 6000 rpm for 30 min. The resulting material was heated to 600 °C under air atmosphere for gelatin removal. Then, the obtained powders were heated to 1000 °C to improve h-BNNS crystallinity. Finally, any remaining impurities in the obtained h-BNNS were removed with several washes in water and ethanol.

### 2.3. Preparation of Membranes

Porous gelatin membranes obtained from h-BNNS-stabilized Pickering emulsions were prepared following a multi-step procedure. Exfoliated h-BNNS (2 wt%) was suspended in water and sonicated with an ultrasound device for 1 h (65% amplitude with pulse off/on 0.5–1 s) to ensure good dispersion. Then, the solution was heated to 60 °C under magnetic stirring and gelatin powder (20 wt%) was added slowly, to avoid lump formation, and kept for 2 h to ensure the complete polymer dissolution. Castor oil (oil/water ratio = 0.8) was added to the as-prepared h-BNNS/gelatin suspension. After sonication for 7 min (45% amplitude and pulse off/on 2–9 s) a homogeneous whitish emulsion was obtained. Films (400 µm of thickness) were prepared at 37 °C by dry-casting the h-BNNS/gelatin solution on flat Teflon-coated sheets, using an automatic coater (K Control coater, Erichsen). Gelatin membranes were cured at 20 °C under 45 ± 5% relative humidity for different drying times (1, 3 and 5 h). This “curing time” allows the system to set. Then, membrane cross-linking was performed by direct immersion in 0.5% GTA solution at 37 °C for different times (1, 3, 5, and 12 h; i.e., “cross-linking time”). The GTA solution was prepared using a 25% GTA stock solution and 96% ethanol as solvent. This step also allows the castor oil extraction. Then, membranes were washed in ethanol and water. These membranes are denoted as BNG-*x* h-*y* h (*x* = drying time in hours, and *y* = cross-linking time in hours).

### 2.4. Membrane Characterization

#### 2.4.1. Membrane Morphology

Scanning Electron Microscopy (SEM) (Hitachi S-4500, Tokyo, Japan) was used to analyze the surface and cross-section morphology of the obtained membranes. After drying at room temperature, membranes were cut in small pieces and sputtered with a thin layer of Pt/Pd before SEM analysis. For cross-section morphology analysis, cut samples were first immersed in liquid nitrogen for 2 h, then broken and dried in a freeze dryer (FreeZone Triad Freeze Dry System 4.5, Labconco Corporation, Kansas City, MI, USA ) for 1 day to obtain a clear break that respects the original structure.

#### 2.4.2. Water Contact Angle

The membrane surface hydrophilicity and wetting characteristics were analyzed by measuring the water contact angle (WCA). WCA values are inversely correlated with the membrane hydrophilicity. Membranes were washed with Milli-Q water, dried overnight and stored in a desiccator until WCA measurement using the sessile drop method. Briefly, after depositing a 10 μL Milli-Q water droplet on the membrane surface using a 50 μL syringe, the drop contour was analyzed using the ImageJ software (Bethesda, MD, USA). The WCA value was calculated with interpolation method and the Drop Snake plugin for ImageJ. For each membrane, the mean value of 10 measurements is shown.

#### 2.4.3. Swelling Ratio

The membrane water solubility was investigated using 2 × 2 cm^2^ samples of the cross-linked films that were desiccated at 50 °C for 48 h. After weighting, samples were put in 30 mL of demineralized water at room temperature, and their swelling kinetics was monitored at different time points by weighting the samples on a micro-balance (Sartorius CPA225D, accuracy of 0.00001 g), after gently blotting with a tissue to remove the excess water. The swelling ratio (SR) was calculated as follows:

(1) $$\mathrm{SR}\left(\%\right)=\frac{\mathrm{weight}\;\mathrm{film}\;-\mathrm{weight}\;\mathrm{dry}\;\mathrm{film}}{\mathrm{weight}\;\mathrm{dry}\;\mathrm{film}}\times 100$$

#### 2.4.4. Pore Size Determination

Pore size was assessed with a PRM-2000-LL-R porometer (IFTS, Foulayronnes, France). Briefly, in each sample, pores were filled with water (wetting step) and then nitrogen flow rate through the membrane was determined as a function of the applied pressure. When the nitrogen flow-applied pressure curve is a straight line, all pores are open. The pore size profile can be calculated using Laplace’s law (2):

(2) $$\Delta P=(4\gamma \cos\theta )/d_{p}$$

where Δ*P* is the differential of the applied pressure, γ is the water interfacial tension, θ is the contact angle between sample and liquid, and d_p_ is the pore diameter. Data are the mean value of three replicates.

### 2.5. Membrane Performances

#### 2.5.1. Filtration Tests

For frontal filtration experiments, a stirred dead-end cell (Amicon 8050, Millipore Corporation) and membranes with an active surface area of 2.27 cm^2^ were used, as previously described [47]. After membrane compaction by filtering pure water at 4 bars until a constant flux was observed, the flux of pure water (J_pw_) of each sample was calculated by circulating deionized water through the membrane system by applying a pressure between 0.5 and 3 bars.

J_pw_ (L h^−1^ m^−2^) was calculated using the following Equation:

(3) $$J_{\mathrm{pw}}=\frac{Q}{\Delta t\;A}=L_{p}\times \Delta P$$

where Q (L) is the amount of collected water, Δ*t* (h) the time duration using a membrane with surface area A (m^2^), L_p_ (L h^−1^ m^−2^ bar^−1^) is the aqueous solution permeability, and Δ*P* (bar) is the transmembrane pressure. The pure water permeability (L_p_) was calculated from the J_pw_ slope in function of the applied pressure.

#### 2.5.2. Polystyrene Latex Particle Rejection Test

The membrane pore sizes were also calculated using polystyrene latex particle rejection tests. A stirred dead-end cell (Amicon 8050, Millipore Corporation, Burlington, MA, USA) and membranes with an active surface area of 2.27 cm^2^ were used for frontal filtration experiments. As before, membranes were first compacted by filtering pure water up to 4 bars until the flux was constant. Then, the feed solution (10 mL ultrapure water that contained polystyrene latex nanoparticles of 0.3 and 1 µm in dimeter) was added to the cell and forced to pass through the membranes by applying a pressure of 3 bars. After filtration, the permeate solution and feed solution were collected and analyzed by dynamic light scattering to determine the amount of nanoparticles that passed through the membrane. Particle rejection was calculated with Equation (4):

(4) $$R\left(\%\right)=(1-\frac{I_{p}}{I_{F}})*100$$

where I_p_ is the intensity of the permeate solution counts and I_F_ is the intensity of the feed solution counts. Data are the mean value of three replicates. After each test, membrane permeability was checked.

#### 2.5.3. Complexation–Ultrafiltration Procedure

Frontal ultrafiltration experiments were performed in a stirred dead-end cell using BNG-5 h-12 h membranes and 100 mL of FeCl_2_·4H_2_O with a feed concentration of 50 ppm and a transmembrane pressure between 0.5 and 3 bars at 25 °C. These solutions were re-circulated in the system by recycling both permeate and retentate. At the steady state, 5 mL of sample was collected. Iron ion concentration in the feed and permeate streams were determined by atomic absorption spectroscopy with an Analyst 400 Perkin Elmer atomic absorption spectrophotometer. The temperature of the feed solution (25 °C) and the transmembrane pressure (3 bars) were constant during the experiments in function of the pH. The pH of feed and permeate solutions was measured with a pHmeter (HI 5221, HANNA Instruments France, Tanneries, France). The efficiency in removing iron ions from the feed solution was determined by calculating the rejection percentage, R_Fe_ (%), with the following Equation:

(5) $$R_{\mathrm{Fe}}\left(\%\right)=\left(1-\frac{C_{p}}{C_{f}}\right)*100$$

where C_p_ and C_f_ are the metal ion concentrations (mg L^−1^) in the permeate and feed solution, respectively.

## 3. Results

### 3.1. Gelatin Membrane Fabrication and Characterization

**Figure 1:**
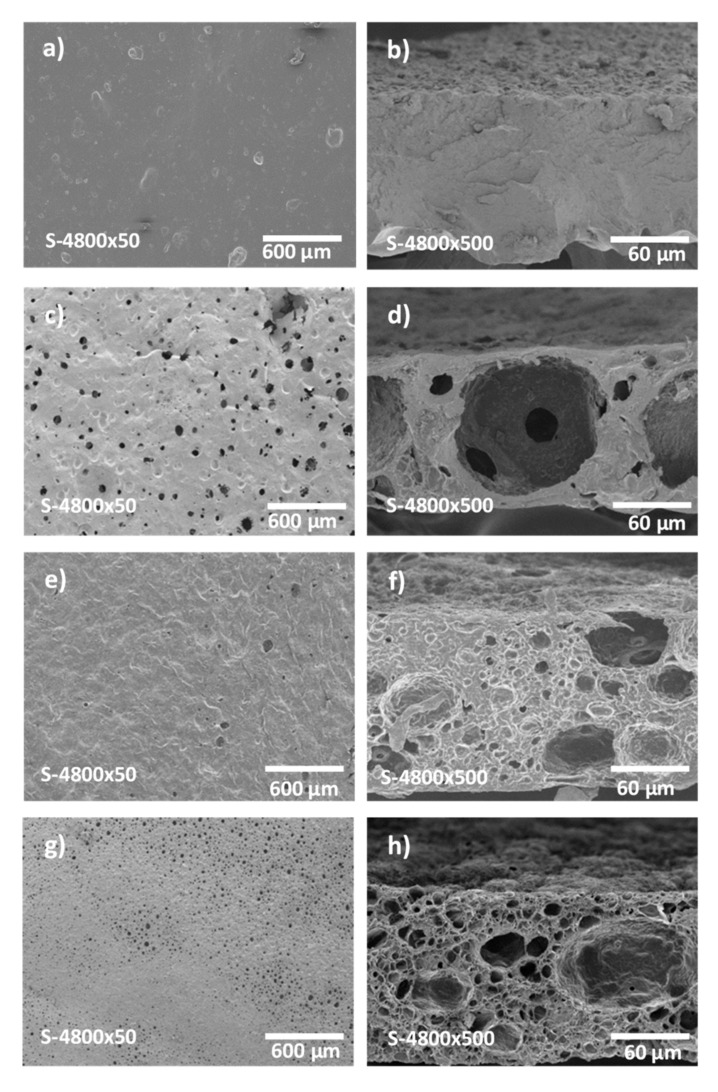
SEM images of the hexagonal boron nitride nanosheets (h-BNNS)/gelatin porous membranes depending on the drying time: (**a**) Surface of free h-BNNS (**b**) cross-section of free h-BNNS (**c**) surface of BNG-1 h-12 h, (**d**) cross-section of BNG-1 h-12 h, (**e**) surface of BNG-3 h-12 h, (**f**) cross-section of BNG-3 h-12 h, (**g**) surface of BNG-5 h-12 h, (**h**) cross-section of BNG-5 h-12 h.

SEM analysis of the morphology of gelatin and h-BNNS/gelatin membranes showed that gelatin membranes were dense, without any sign of porosity in their internal structure and at their surface (Figure 1). Conversely, h-BNNS/gelatin membranes displayed porosity in cross-sections and also at their surface. Specifically, membrane porosity was correlated with the curing time length (Figure 1). Membranes cured for longer times presented smaller pores in their internal structure and also at their surface. The pores of BNG-5 h-12 h membranes were smaller than those of BNG-3 h-12 h and BNG-1 h-12 h membranes. The reduced porosity observed after 5 h of curing might be explained by two phenomena. Due to the slow water evaporation during curing, the gelatin chains come closer to each other. Concomitantly, the temperature reduction after membrane casting at 60 °C and during the drying step at 20 °C leads to gelation of the continuous phase. This hinders the coalescence of the oil phase droplets that may occur during membrane immersion in the cross-linking bath in the next step of the membrane preparation procedure. Emulsion droplet coalescence generates bigger cells, as observed on the SEM micrographs of the membrane cross-sections (Figure 1d). The surface membrane porosity (when drying time was set at 5 h) was also influenced by the cross-linking time. The surface pore size was smaller in BNP-5 h-12 h than in BNP-5 h-1 h, BNP-5 h-3 h, and BNP-5 h-5 h membranes. This is likely to be due to rupture of inter- and intra-molecular hydrogen bonds between water and gelatin induced by the ethanol present in the cross-linking solution. This leads to the gelatin chain reorganization through (i) neutralization of selective charges, and (ii) entropy gain due to the random mixing of gelatin and the release of counter ions.

### 3.2. WCA Measurements

**Figure 2:**
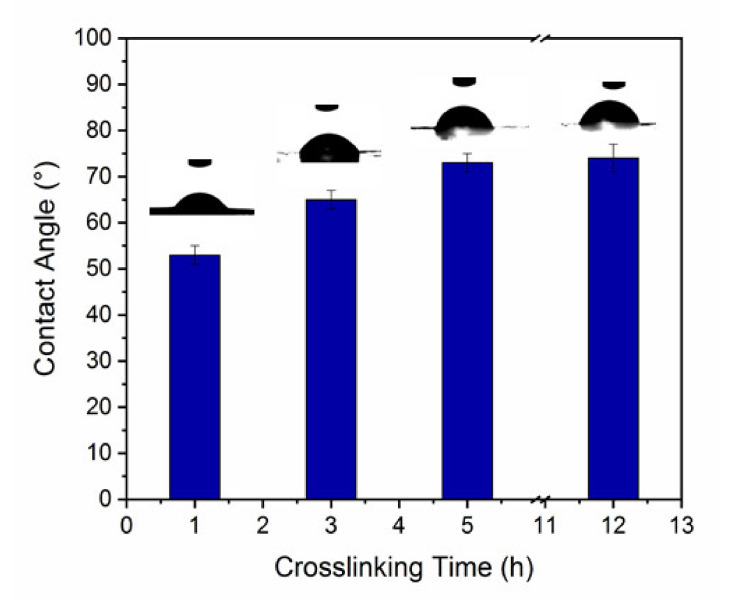
Water contact angles of h-BNNS/gelatin membranes cured for 5 h and cross-linked for different lengths of time.

WCA measurements were carried out to characterize the membrane hydrophilic behavior. Figure 2 shows the water droplets at the surface of h-BNNS/gelatin membranes that were cross-linked for different lengths of time (photographs taken during the first 5 s after deposition of the water droplet on the membrane surface). WCA values were higher for gelatin membranes (86 ± 2°) compared with h-BNNS/gelatin membranes cross-linked for 1 h (52 ± 2°). WCA tended to increase with the cross-linking time. Indeed, membranes cross-linked for 3 h, 5 h, and 12 h displayed contact angles of about 64 ± 2°, 72 ± 2°, and 74 ± 3°, respectively. After the first 5 s, the water droplet spread and rapidly penetrated in the membrane, resulting in a WCA decrease to approximately 0°, except for the BNP-5 h-12 h membrane where droplet absorption took longer. The cross-linking method influences membrane hydrophobicity. When crosslinking is done using GTA, the polymer amine groups react with GTA aldehyde groups, leading to lower hydrophilicity [34]. However, WCA differences among gelatin membrane and h-BNNS/gelatin membranes cross-linked for different lengths of time is also related to their different surface porosity (Figure 1) that allows faster water absorption compared with gelatin membranes that have a non-porous surface. In the case of BNP-5 h-12 h, the longer absorption time might be due to the presence of smaller holes at the surface, as observed in the SEM images. Therefore, it is important to note that chemical cross-linking modifies the membrane network structure, significantly reducing the pore size distribution.

### 3.3. Swelling Ratio

**Figure 3:**
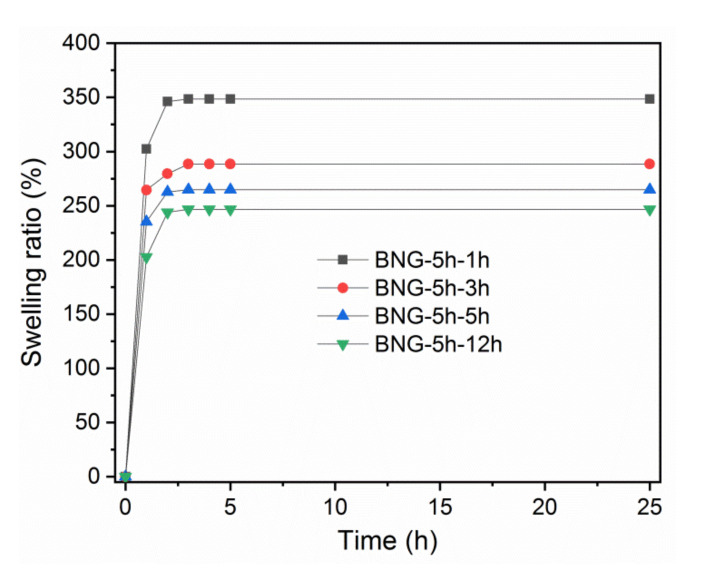
Swelling ratio of h-BNNS/gelatin membranes cross-linked for different lengths of time.

As gelatin is water-soluble, crosslinking with GTA is essential for the long-term use of h-BNNS/gelatin membranes. However, the GTA-based cross-linking time influenced the membrane porosity, and this could affect their swelling behavior. Measurement of the swelling capacity of h-BNNS/gelatin membranes (cross-linked for different lengths of time) in water (Figure 3) suggested that their swelling patterns were comparable. Indeed, swelling was fast at the beginning and then progressively decreased to reach a constant value at 3 h. The SR of the four membranes ranged between 247 and 348%, and was higher for the BNG-5 h-1 h membrane compared with the BNG-5 h-3 h, BNG-5 h-5 h, and BNG-5 h-12 h membranes. This could be explained again by the differences in surface porosity of the four membranes and also by the cross-linking effectiveness. Indeed, water absorption is faster and water absorption capacity is higher for membranes with higher porosity. Moreover, as longer cross-linking with GTA reduces the hydrophilicity and the polymer chain mobility, the swelling capacity of membranes cross-linked for longer time is reduced.

### 3.4. Pore Size Determination

**Figure 4:**
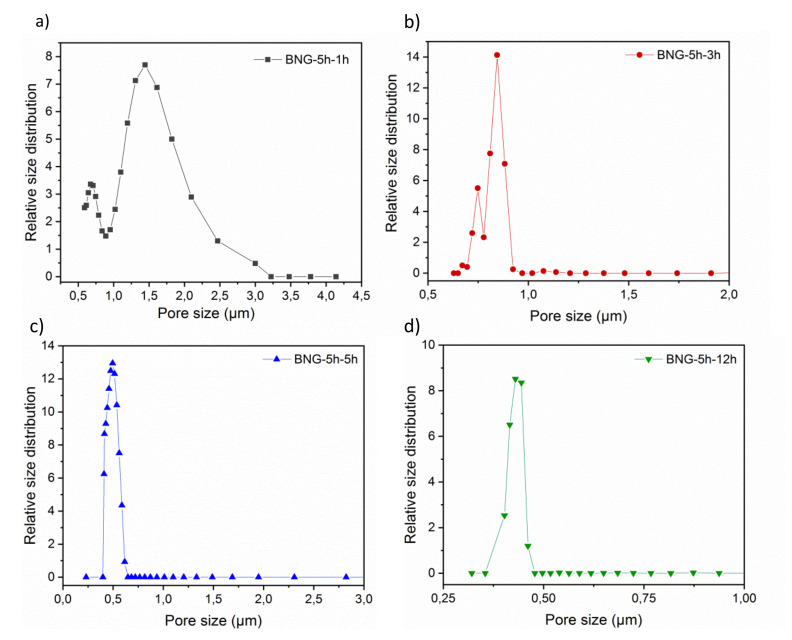
Relative pore size distribution (determined by gas–liquid displacement porometry) of h-BNNS/gelatin porous membranes in function of the cross-linking time. (**a**) BNG-5 h-1 h, (**b**) BNG-5 h-3 h, (**c**) BNG-5 h-5 h, and (**d**) BNG-5 h-12 h membranes.

The gas–liquid displacement porometry analysis allowed assessing the membrane mean pore diameter and the pore size distribution at the surface. The pore size distribution of the BNG-5 h-1 h, BNG-5 h-3 h, BNG-5 h-5 h, and BNG-5 h-12 h membranes (Figure 4) was in agreement with the SEM finding that the mean pore size is smaller in the BNG-5 h-12 h than in the BNG-5 h-5 h, BNG-5 h-3 h, and BNG-5 h-1 h membranes. Specifically, the mean pore size was 0.39 ± 0.03 μm for the BNP-5 h-12 h membrane, 1.60 ± 0.03 μm for the BNP-5 h-1 h membrane, 0.84 ± 0.03 μm for the BNP-5 h-3 h, and 0.49 ± 0.03 µm for the BNP-5 h-5 h membrane. This confirmed that the membrane morphology and pore size distribution are strongly influenced by the cross-linking time, as previously reported for polymer-based membrane structures [48,49,50]. With these pore size distributions, these membranes might be used for microfiltration or ultrafiltration purposes.

### 3.5. Membrane Performances

#### 3.5.1. Filtration

**Figure 5:**
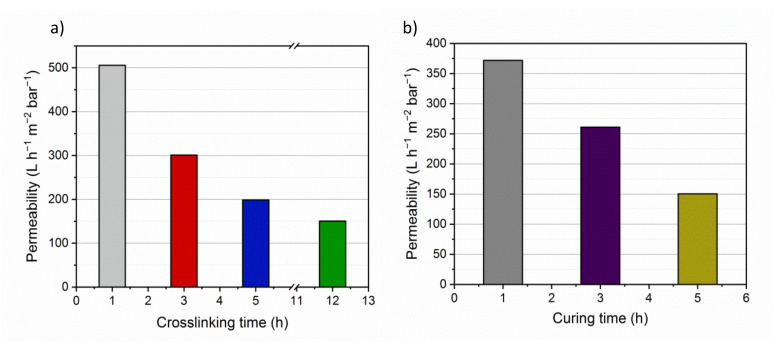
Pure water permeability of h-BNNS/gelatin porous membranes that underwent (**a**) cross-linking and (**b**) curing for the indicated lengths of time.

L_p_ value is directly related to the membrane porosity, pore size and surface hydrophilicity. The flux is influenced by the membrane material resistance in the presence of a specific differential pressure across the membrane. Therefore, the flux increases with the membrane operating area and the applied pressure. Analysis of the membranes cross-linked for different lengths of time (Figure 5a) showed that the L_p_ value was higher for the BNG-5 h-1 h membrane (506 L h^−1^ m^−2^ bar^−1^) than for the BNG-5 h-3 h, BNG-5 h-5 h, and BNG-5 h-12 h membranes (301, 199, and 150 L h^−1^ m^−2^ bar^−1^, respectively). In membranes cured for different lengths of time (Figure 5b) the L_p_ value was higher for the BNG-1 h-12 h membrane (372 L h^−1^ m^−2^ bar^−1^) than for the BNG-3 h-12 h and BNG-5 h-12 h membranes (261 and 150 L h^−1^ m^−2^ bar^−1^, respectively). These findings demonstrates that cross-linking and curing times influence the membrane pore size, and consequently the membrane water-filtering performance because higher porosity leads to higher water permeability flux. The permeability values match with SEM observations (Figure 1), the membrane BNG-1 h-12 h presents larger pores, both in surface and in the cross-section SEM view leading to higher permeability values. On the other hand the membranes BNG-3 h-12 h and BNG-5 h-12 h display smaller pores either in surface and cross-section SEM micrograph leading to lower permeability values. The results also agree with the obtained pore size distribution of each membrane: Larger pores lead to higher permeability values. The water flux obtained in this work is higher than the flux obtained by gelatin-based membranes already presented in the literature. Polyamide (PA) thin film nanofibrous composite membranes with a polyacrylonitrile (PAN) substrate and gelatin interlayer fabricated by reverse interfacial polymerization (IP-R) display a flux of 135.6 L m^−2^ h^−1^ [51], whereas gelatin membranes previously prepared in our group using Pickering emulsion method but with graphene oxide (GO) as stabilizer and ethyl benzoate as oil phase were characterized with a quite low water permeability of 5.8 ± 1.3 L m^−2^ h^−1^bar^−1^ [52]. The complexation–ultrafiltration tests were carried out using the BNG-5 h-12 h membrane, and its L_p_ value (150 L h^−1^ m^−2^ bar^−1^) was considered the reference value to assess concentration polarization and membrane fouling. The L_p_ value was calculated before each test.

#### 3.5.2. Polystyrene Latex Particle Rejection Test

The ability of the BNG-5 h-12 h membrane to work as ultrafiltration membrane was assessed using a particle rejection test in which the filtration cell was filled with 0.3 or 1 μm polystyrene latex nanoparticles that were forced to pass through the membrane by applying a pressure of 3 bars. Quantification of the BNG-5 h-12 h membrane rejection percentages for the two nanoparticle sizes showed that it could reject 10 ± 2% and 96 ± 1% of 0.3 and 1 μm latex nanoparticles, respectively. This finding can be explained by the fact that the BNG-5 h-12 h membrane mean pore size was 0.39 ± 0.03 μm. Some nanoparticles of 0.3 m actually could get into the porous membrane because many surface pores were larger than 0.3 μm as illustrated on Figure 4d and they can easily travel inside the membranes due to the pores might be connected facilitating their transport.

#### 3.5.3. Complexation–Ultrafiltration Procedure

##### Ultrafiltration of An Iron Ion Solution

**Figure 6:**
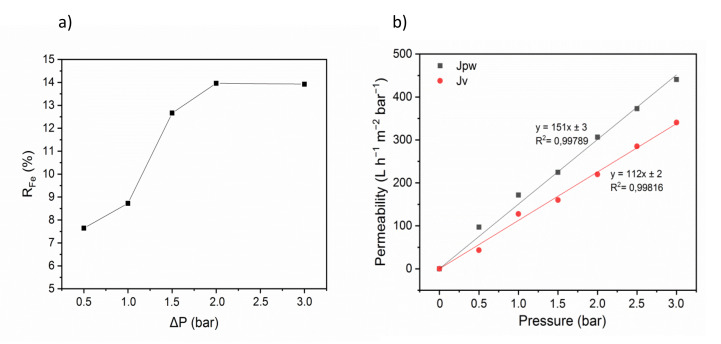
(**a**) Iron retention in function of the transmembrane pressure, [Fe^2+^] = 50 ppm, θ = 25 °C; and (**b**) Flux of pure water (J_pw_) and of the aqueous iron solution permeate (J_v_) in function of the transmembrane pressure, θ = 25 °C and [Fe^2+^] = 50 ppm.

Analysis of iron retention changes in function of the transmembrane pressure (Δ*P*) showed that iron ion retention was low and did not exceed 14% (Figure 6a). Nevertheless, this indicates that some iron ions might be adsorbed onto gelatin through electrostatic interactions. Therefore, the use of a polyelectrolyte as complexing agent is a relevant strategy.

Quantification of the flux of pure water (J_pw_) and of the aqueous iron solution permeate (J_v_) in function of the transmembrane pressure showed that J_pw_ was proportional to the transmembrane pressure, as predicted by Equation (3) (Figure 6b). The slope represents the membrane permeability to pure water (L_p_ = 150 L h^−1^ m^−2^ bar^−1^) and to the aqueous iron solution (L_p_ = 113 L h^−1^ m^−2^ bar^−1^). This result suggests that the addition of the iron ion solution does not significantly increase resistance, generally observed when solutes are filtered by the membrane and related to the capture of a part of iron ions in the membrane by adsorption. The iron ion solution permeability was higher for the membranes fabricated in this work compared with the values obtained using commercial poly-sulfone membranes and polyvinyl alcohol (PVA) as complexing agent (113 L h^−1^ m^−2^ bar^−1^ vs 15 L h^−1^ m^−2^ bar^−1^) [53]. Our membranes displayed also higher permeability compared with commercial polyether sulfone membranes (75 L h^−1^ m^−2^ bar^−1^ with 25 ppm of aqueous metal solution) [54].

##### Ultrafiltration of an Iron Ion Solution in the Presence of PAA

Effect of Transmembrane Pressure and Polymer Concentration

The formation of polymer-metal complexes is mainly due to electrostatic forces and the formation of coordinating bonds. The metal ions in the cell can appear in three configurations: Free in the solution or unbounded, bound to the polymer and linked to the membrane due to rejection and/or sorption [55]. As shown previously the metal ions rejections are low. In order to ameliorate these rejections, it was interesting to add a polyelectrolyte as complexing agent. In this study, we choose the PAA as chelating agent. The principle of complexation–ultrafiltration (PAUF) is based on a sequence of steps with a complexation of iron ions by a complexing polymer (PAA) and a step involving the rejection of the complex formed by means of a UF membrane. Unlike free ions which cross the membrane, the macroligand-ion complex (PAA-Fe) is held back.

This hybrid process generates one solution that is purified and one that has a high concentration of complexes. Two kinds of metal ions, both smaller than the membrane pore diameter, are defined: Free metal ions in solution, whose movement across the membrane is not restricted and metals bound to the polymer which cannot cross the membrane. It is worth mentioning that in PEUF processes the retention of a species of interest mainly depends on its interaction with the macromolecular chains and is independent of its size.

**Figure 7:**
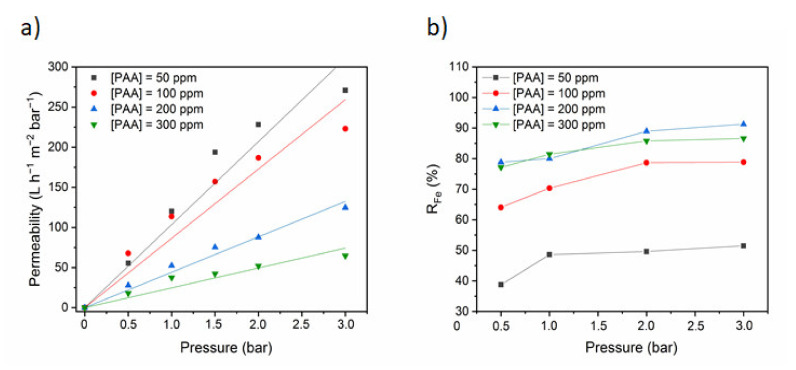
(**a**) Permeate flux in function of the transmembrane pressure between 0.5 and 3 bars at 25 °C in the presence of the indicated poly (acrylic acid) (PAA) concentrations and [Fe^2+^] = 50 ppm; and (**b**) Iron retention in function of the transmembrane pressure in the presence of the indicated PAA concentrations, [Fe^2+^] = 50 ppm.

Figure 7a shows the quantification of the permeate flux in the presence of Fe^2+^ and of different transmembrane pressures (Δ*P*) and PAA concentrations. The permeate flux was higher with higher transmembrane pressures, whereas it was reduced in the presence of progressively higher PAA concentrations. This decrease might be explained by membrane fouling and concentration polarization. Indeed, at pH 6 the carboxylic groups of PAA are almost totally dissociated and the chains of PAA should be extended due to the repulsion of their carboxylate groups. Furthermore, at low ion concentrations, the electrostatic factors dominate the adsorption phenomena of the polyelectrolyte on the surface leading to a decrease on the permeability values [55,56]. The osmotic pressure gradient also might influence ultrafiltration of metal ions in the presence of PAA. The flux variations in function of the transmembrane pressure did not follow Darcy’s law; however, the permeate flux was reduced in the presence of PAA compared with the values obtained when using only the iron ion solution. This reduction is explained by the higher viscosity of the PAA-iron ion solutions compared with pure water. Indeed, ultrafiltration membranes are permeable to water and iron ions, but not to polyelectrolytes. Moreover, the non-homogeneous iron ion distribution generates osmotic and swelling pressures between phases [57].

The adsorption of polyelectrolytes onto a surface is governed by a number of parameters such as: The polyelectrolyte and surface charge densities and their sign, the ionic strength and the pH of the solution. Transmembrane pressure and PAA concentration also influenced iron ion retention (Figure 7b). Indeed, the iron retention percentage progressively increased with PAA concentration up to 200 ppm PAA (R_Fe_ = 91%), and then decreased. This can be explained by different factors, including concentration polarization, membrane fouling, precipitation, and formation of a gel layer on the membrane surface. For example, at the beginning, the transmembrane pressure is low and carboxylate groups of PAA are available to a possible interaction with metal ions. As the transmembrane pressure increases the number of available sites decreases as they are being occupied. A maximal R_Fe_ would be reached when the maximum capacity of the polymer is attained. We can consider that after 200 ppm of PAA, the maximum capacity is reached, and the retention capacity starts to decrease. This fact could be due to the modifications in the association capacity of the polymer possibly due to conformational changes induced in the polymer [55]. The iron retention increases with the increase of PAA concentration from 50 to 200 ppm, until reaching 91%. Beyond this concentration, retention of Fe^2+^ decreases. This is mainly attributed to several phenomena, such as concentration polarization, membrane fouling, osmotic pressure, precipitation, and the formation of a gel layer on the membrane surface. These phenomena can be revealed by the reduction of permeate flux (J_v_) as a function of transmembrane pressure [58]. Palencia et al. reported the same behavior and the results were discussed on the bases of a model taking into account different interactions: polymer membrane; membrane-metal ion; polymer-metal ions and polymer–metal complexes–membrane. The authors concluded that the later interaction plays a central role; the strength of this interaction was correlated to the energy of solvation of the cations [55]. Decreasing solvation energies should correspond to stronger interactions and easier complexation with the polymer [59].

Effect of pH

**Figure 8:**
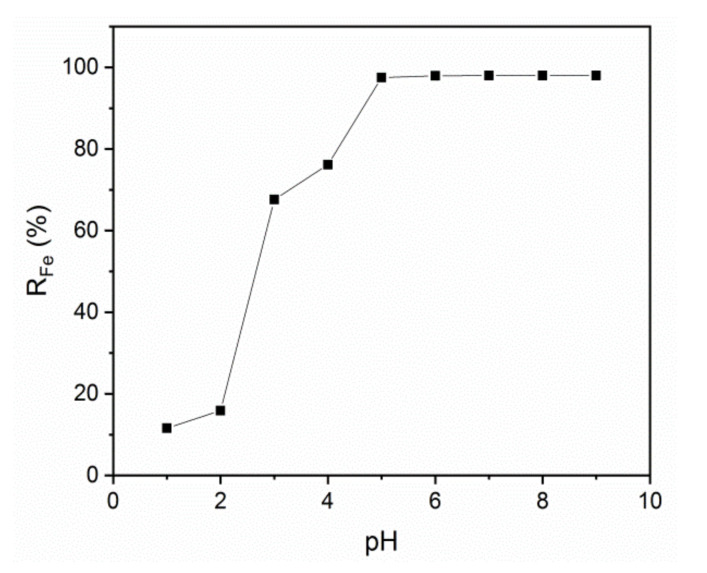
Iron retention in function of the pH, [Fe^2+^] = 50 ppm, [PAA] = 200 ppm, Δ*P* = 3 bars.

pH strongly influences polymer retention by causing aggregation phenomena or repulsion of polymer chains. It can also cause polymer precipitation. Thus, the effect of changing the pH of the feed solution on the membrane iron ion retention efficiency was assessed in the presence of an iron ion concentration of 50 ppm and transmembrane pressure of 3 bars. The membrane iron ion retention efficiency increased with higher pH up to 97% at pH 5 (Figure 8). Higher pH leads to an increase in the concentration of deprotonate carboxylic groups that promotes the formation of macromolecular polymer–metal complexes, and consequently to higher metal retention coefficients [60,61]. The iron retention efficiency of our porous membranes is comparable to that of commercial poly-sulfone and polyether sulfone membranes that can retain 97% of Fe^3+^ ions at pH 7 [53,54]. At lower pH, the polymer carboxylic functional groups do not dissociate and the PAA chains form highly compact clusters that are joined by short extended parts of polymer chains with a microenvironment polarity identical to that of the PAA hydrophobic areas. In the presence of a basic solution, the polymer carboxylic groups start dissociating and carboxylate anions (COO^−^) become more important. The molecule electrical charges lead to the appearance of intramolecular and intermolecular repulsion forces. Moreover, at pH higher than 5, gelatin displays carboxylate anions that can interact with iron ions. The retention of Fe^2+^ at pH 5 using PAA as electrolyte is higher than using another divalent ion, such as Cu^2+^. For example, Magnenet et al. studied the influence of pH on the retention of copper ions using PAA polyelectrolyte-modified membranes. The results showed that at low pH values and transmembrane pressure about 1.8 bar the retention rate of Cu^2+^ was 44% and increasing the pH to 5, the retention rate decrease to 23% [62].

## 4. Discussion

In this work, we prepared porous gelatin membranes using an innovative method based on Pickering emulsion templating stabilized by h-BNNS. Membrane preparation includes two steps that directly affect the membrane morphology: (i) The cast Pickering emulsion is dried at 20 °C and 45 ± 5% of relative humidity; and (ii) membranes are cross-linked in 0.5% GTA/ethanol. Indeed, SEM analysis indicated that the pore size decreased with the increase of the drying and cross-linking times. Cross-linking duration also influences the membrane hydrophobicity. The WCA increased from 52 to 74° when the cross-linking time passed from 1 h to 12 h. Increasing cross-linking duration reduced the membrane hydrophilicity behavior and the pore size at the surface, thus the membrane swelling capacity was decreased. The pore size distribution of the h-BNNS/gelatin membranes, assessed by porometry, was in agreement with the SEM data showing that the mean pore size decreases with longer cross-linking times. BNP-5 h-12 h membranes (5 h of drying and 12 h of cross-linking) had a mean pore size of 0.39 ± 0.03 μm. Pure water permeability (L_p_) is directly correlated with pore size and membrane surface hydrophilicity. In agreement, L_p_ values were higher for membranes with shorter cross-linking and drying times. Thus, our approach allows fabricating membranes with L_p_ between 150 and 506 L h^−1^ m^−2^ bar^−1^ and pore size between 0.39 and 1.60 μm. The use of h-BNNS is essential to maintain the membrane porosity, first through the stabilization of the emulsion droplets along casting, but also once the membranes are fabricated when they are supporting pressure along filtration. In this work, operating conditions of membrane preparation process are rather been investigated. However, nanoparticles with different surface properties could be tested as stabilizer for Pickering emulsion for a better understanding of their influence on the membrane morphology. As perspectives to this experimental study, theoretical and modeling studies could be conducted to have better comprehension of the interactions that h-BNNS might have with iron (II) ions, PAA or the interactions after the complexation of Fe^2+^–PAA [63].

These membranes were tested in PEUF to remove iron ions from aqueous solutions with the aim to depict their behavior under different conditions (pressure, polymer concentration, pH) and compare them with commercial membranes performances. In the absence of PAA, free Fe (II) ion rejection was low and did not exceed 14%. In the presence of PAA, the permeate flux increased when higher transmembrane pressure were applied, and decreased with higher polyelectrolyte concentrations. The highest iron ion retention rates were observed with 200 ppm of PAA and 3 bars of transmembrane pressure (91%) and with a feed solution at pH 5 (97%).

## 5. Conclusions

Pickering emulsion templating represents a novel strategy for the fabrication of promising porous membranes with biodegradable and hydrophilic polymers and for filtration applications, such as PEUF described in this work. Results highlight the possibility to tailor the membrane porosity by the means of a rigorous control of elaboration process parameters. This method is sustainable since it uses mainly water as solvent. The main reagents, gelatin and castor, are low cost and allow to envision the preparation towards this method biodegradable disposal filters.

## Abbreviations

**AgNP**	silver nanoparticles
**BSA**	bovine serum albumin
**CNT**	carbon nanotubes
**GO**	graphene oxide
**GTA**	glutaraldehyde
**h-BNNS**	Hexagonal boron nitride nanosheets
**J_pw_**	flux of pure water
**J_v_**	aqueous iron solution permeate
**L_p_**	pure water permeability
**MW**	molecular weight
**NIPS**	non-solvent induced phase separation
**PAA**	poly (acrylic acid)
**PAN**	Polyacrylonitrile
**PEG**	polyethylene glycol
**PEUF**	polyelectrolyte-enhanced ultrafiltration
**PLA**	Poly (L-lactic acid)
**PSU**	polysulphone
**R_Fe_**	iron ions rejection percentage
**SEM**	Scanning Electron Microscopy
**SR**	swelling ratio
**TIPS**	thermally induced phase separation
**WCA**	Water Contact Angle
